# Non-alcoholic fatty liver disease is associated with bacterial translocation and a higher inflammation response in psoriatic patients

**DOI:** 10.1038/s41598-021-88043-8

**Published:** 2021-04-21

**Authors:** Isabel Belinchón-Romero, Pablo Bellot, David Romero-Pérez, Isolina Herraiz-Romero, Francisco Marco, Rubén Frances, José-Manuel Ramos-Rincón

**Affiliations:** 1 Dermatology Department, University General Hospital of Alicante & Alicante Institute of Sanitary and Biomedical Research (ISABIAL), C/Pintor Baeza, 12, 03010 Alicante, Spain; 2 Clinical Medicine Department, Miguel Hernández University of Elche, Elche, Spain; 3 Digestive Medicine Department, University General Hospital of Alicante & Alicante Institute of Sanitary and Biomedical Research (ISABIAL), Alicante, Spain; 4 Dermatology Unit, Hospital Quirón-Salud, Tenerife, Spain; 5 Radiodiagnostic Department, University General Hospital of Alicante & Alicante Institute of Sanitary and Biomedical Research (ISABIAL), Alicante, Spain; 6 Immunology Department, University General Hospital of Alicante & Alicante Institute of Sanitary and Biomedical Research (ISABIAL), Alicante, Spain; 7 Research Institute, University General Hospital of Alicante & Alicante Institute of Sanitary and Biomedical Research (ISABIAL), Alicante, Spain; 8 CIBERehd, Instituto de Salud Carlos III, Madrid, Spain; 9 Internal Medicine Department, University General Hospital of Alicante-ISABIAL, Alicante, Spain

**Keywords:** Diseases, Skin diseases, Hepatology

## Abstract

Psoriasis and non-alcoholic fatty liver disease (NAFLD) are both inflammatory diseases. The study objective was to estimate the risk of NAFLD, non-alcoholic steatohepatitis, and liver fibrosis (by liver stiffness and liver biopsy) in patients with psoriasis and to determine the epidemiological, clinical, immunological (TNF-α, IL-2, IL-6, IL-12, IL-17, IL-23, and TGF-β) characteristics, and bacterial translocation. Of the 215 psoriatic patients included, 91 presented NAFLD (prevalence: 42.3%). Compared to patients with psoriasis alone, those with NAFLD were significantly more likely to have metabolic syndrome, diabetes, dyslipidemia, body mass index ≥ 30 kg/m^2^, homeostatic model assessment of insulin resistance ≥ 2.15, and greater psoriasis area severity index. NAFLD patients also had significantly higher levels of TNF-α (p = 0.002) and TGF-β (p = 0.007) and a higher prevalence of bacterial translocation (29.7% vs. 13.7%; p = 0.004). Liver stiffness measurement was over 7.8 kPa in 17.2% (15/87) of NAFLD patients; 13 of these underwent liver biopsy, and 5.7% (5/87) had liver fibrosis, while 1.1% (1/87) had advanced fibrosis or non-alcoholic steatohepatitis. In conclusion the prevalence of NAFLD in patients with psoriasis is high and associated with a higher prevalence of metabolic syndrome features, bacterial translocation and a higher pro-inflammatory state. It is worth mentioning that liver fibrosis and non-alcoholic steatohepatitis are not frequent in this population of patients.

## Introduction

Psoriasis is a systemic, immune-mediated genetic disease associated with several comorbidities (particularly in severe forms)^1–5^; indeed, over the last years several studies have been shown that non-alcoholic fatty liver disease (NAFLD) to be highly prevalent in patients with psoriasis^6,7^. The term NAFLD encompasses a wide spectrum of hepatic lesions, ranging from simple fatty liver to non-alcoholic steatohepatitis (NASH), and including variable degrees of liver fibrosis, cirrhosis, and even hepatocellular carcinoma^8,9^. The global prevalence of NAFLD in the general population is estimated to be 25%^10^, and it is currently one of the leading causes of cirrhosis and liver transplantation^11^. Nowadays, NAFLD is a growing epidemic, in part due to obesity, insulin resistance, and metabolic syndrome^12^, but also due to psoriasis^13^.

It is striking that the same comorbidities, especially those associated with metabolic disorders that can promote liver steatosis, have been associated with systemic inflammation in psoriasis. Moreover, specific proinflammatory mediators have been shown to cause a chronic inflammatory state in NAFLD, psoriasis, and metabolic syndrome^14,15^. These similarities could indicate a linked pathogenesis between psoriasis and NAFLD, with a potentially increased risk for advanced hepatic disease^12^. However, controversy remains around whether the chronic inflammatory nature of psoriasis is a contributing factor or an independent risk factor for the development of NAFLD.

Dysregulation of immune responses in individuals who are genetically susceptible to psoriasis and have been exposed to an external environmental trigger is essential in plaque psoriasis pathogenesis^16–18^. Various cytokines (tumor necrosis factor [TNF]-α, type I interferons [IFNs], interleukin [IL]-12, IFN-γ, IL-23, IL-17, and IL-22) mediate the interaction of keratinocytes, dendritic cells, T cells, and other immune cells, causing an abnormal loop proliferation of keratinocytes in the psoriatic epidermis^15^. Cytokines are elements of immunity that mediate the inflammatory response of the psoriasis plaque and play a mechanistic role in the development of insulin resistance and fatty liver disease. However, their role as biomarkers of NAFLD in psoriatic patients is not well established^19,20^. Previous studies have suggested that NAFLD is associated with an increased pro-inflammatory state related to abnormal intestinal permeability, endotoxemia and bacterial translocation^21,22^, so the NAFLD pro-inflammatory profile could be associated with psoriasis.

The aim of this study was to estimate the risk of NAFLD, NASH, and liver fibrosis in patients with psoriasis and to determine the epidemiological, clinical, immunological, and inflammatory characteristics, along with the rate of bacterial translocation, in these patients.

## Results

An initial sample of 309 patients with moderate to severe psoriasis were registered during the recruitment period. After excluding 79 patients who presented at least one exclusion criterion, 13 who did not complete the required testing, and 2 who did not have available cytokines, we finally included 215 patients (120 men and 95 women; Supplementary Fig. S1).

### Prevalence and risk factors of NAFLD

Ninety-one (42.3%) included patients presented NAFLD: 62 cases were mild, and 29 were moderate to severe. Patients with and without NAFLD were similar in terms of the treatments they were receiving for psoriasis, except for acitretin, which was more common in patients with NAFLD (11.0% vs 3.2%; p = 0.02; Supplementary Table S1).

NAFLD was associated with male sex; older age; body mass index (BMI) of 30 kg/m^2^ or more; homeostatic model assessment of insulin resistance (HOMA-IR) of more than 2.15; diabetes mellitus; cardiopathy; dyslipidemia; metabolic syndrome; higher waist circumference, levels of aspartate aminotransferase (AST), alanine aminotransferase (ALT), gamma-glutamyl transferase (GGT), LDL-cholesterol, triglycerides, erythrocyte sedimentation rate (ERS), and high-sensitivity C-reactive protein (hs-CRP); and non-viable bacterial translocation (BT). Smoking was more prevalent in psoriatic patients without NAFLD. Patients with psoriatic arthritis did not present more NAFLD (Table 1).

**Table 1:** Demographic, clinical, and laboratory characteristics of psoriatic patients with and without non-alcoholic fatty liver disease (NAFLD).

Variable	No NAFLD (n = 124)	NAFLD (n = 91)	P value
**Demographics and laboratory**			
Men, n (%)	61 (49.2)	59 (64.8)	0.023
Age in years, median (IQR)	41.5 (35–51)	53 (45.5–61.5)	< 0.001
Years of disease evolution, median (IQR)	16 (10–23.5)	20 (10–28)	0.20
BMI ≥ 30 kg/m^2^, n (%)	26 (21.0)	54 (59.3)	< 0.001
Abnormal waist circumference*, n (%)	71 (90.1)	8 (90.1)	< 0.001
Smokers, n (%)	50 (40.3)	23 (25.3)	0.021
HOMA-IR ≥ 2.15, n (%)^†^	57 (47.5)	83 (91.2)	< 0.001
Diabetes mellitus, n (%)	8 (6.5)	23 (25.3)	< 0.001
Cardiopathy, n (%)	5 (4.0)	10 (11.0)	0.048
Dyslipidemia, n (%)	45 (36.3)	57 (62.6)	< 0.001
Metabolic syndrome, n (%)	30 (24.2)	54 (59.3)	< 0.001
Psoriasis arthritis, n (%)	18 (14.5)	19 (20.9)	0.22
AST > 32 U/L, n (%)	4 (3.2)	14 (15.4)	0.001
ALT > 33 UL, n (%)	14 (11.3)	29 (31.9)	< 0.001
GGT > 40 U/L, n (%)	19 (15.3)	31 (34.1)	0.001
Cholesterol > 200 mg/dL, n (%)	33 (26.8)	33 (36.3)	0.13
LDL-cholesterol > 100 mg/dL, n (%)	90 (72.6)	78 (85.7)	0.021
Triglycerides > 150 mg/dL, n (%)	20 (16.1)	40 (44.0)	< 0.001
Albumin < 3.5 g/dL, n (%)	2 (1.6)	1 (1.1)	0.99
Hemoglobin < 11.5 g/dL, n (%)	0 (0.0)	1 (0.8)	0.99
White blood cells < 4.5 × 10^9^/L, n (%)	5 (4.0)	1 (1.1)	0.45
Platelets < 150 × 10^9^ /L, n (%)	6 (4.8)	1 (1.1)	0.43
ERS ≥ 16 mm, n (%)	34 (28.1)	33 (36.3)	0.28
Hs-CRP ≥ 0.1 mg/mL, n (%)	82 (67.2)	81 (89.0)	< 0.001
Non-viable BT, n (%)	17 (13.7)	27 (29.7)	0.004
**Severity scale**			
Psoriasis Area and Severity Index (PASI), median (IQR)	1.5 (0–5)	3.4 (1–8)	0.008
Body surface area (BSA), median (IQR)	2 (0–6)	4 (1–8)	0.007
Physician global assessment (PGA) (range 0–4), median (IQR)	1 (0–2)	2 (1–3)	0.004
Dermatology Life Quality Index (DLQI), median (IQR)	2 (0–5)	2 (1–3)	0.74
Itch, visual analog scale (range 0–10), median (IQR)	2 (0–6)	3 (0–6)	0.52
Pain, visual analog scale (range 0–10), median (IQR)	0 (0–6)	0 (0–6)	0.23

*AST* aspartate aminotransferase, *ALT* alanine aminotransferase, *BMI* body mass index, *BT* bacterial translocation, *ERS* erythrocyte sedimentation rate, *GGT* g-glutamyl transferase, *IQR* interquartile range, *hs-CPR* high-sensitivity C-reactive-protein, *HOMA-IR* homeostatic model assessment of insulin resistance, *LDL* low-density lipoprotein.

*Abnormal waist circumference: women, ≥ 86 cm, ≥ 95 cm male.

^†^HOMA-IR available in 211 patients.

Multivariable logistic regression analysis confirmed that HOMA-IR over 2.15 (p < 0.001), male sex (p = 0.005) and BMI of 30 kg/m^2^ or more (p = 0.007), AST more than 32 U/L (p = 0.036), and triglycerides of more than 150 mg/dL (p = 0.026) significantly increased the risk of NAFLD (Table 2).

**Table 2:** Risk of developing non-alcoholic fatty liver disease (NAFLD), according to explanatory variables (bivariable and multivariable analysis).

Variable	Crude OR (95% CI)	Adjusted OR (95% CI)	P value
Sex (male)	1.90 (1.09–3.32)	3.36 (1.44–7.87)	0.005
Age	1.05 (1.03–1.08)	0.98 (0.94–1.02)	0.36
Years of evolution of psoriasis	1.02 (0.96–1.09)	–	
BMI ≥ 30 kg/m^2^	6.08 (3.14–14.74)	3.47 (1.40–8.85)	0.007
Abnormal waist circumference*	5.56 (2.72–11.56)	1.06 (0.32–3.56)	0.92
Smokers	0.51 (0.28–0.90)	0.58 (0.24–1.42)	0.23
HOMA-IR ≥ 2.15^†^	11.46 (5.10–25.7)	7.75 (1.30–22.72)	< 0.001
Diabetes mellitus	4.90 (2.09–11.57)	2.85 (0.85–9.61)	0.090
Cardiopathy	2.94 (0.97–8.91)	1.25 (0.23–6.75)	0.78
Dyslipidemia	2.94(1.68–5.2)	1.14 (0.37–2.60)	0.88
Metabolic syndrome	4.57 (2.54–8.22)	1.05 (0.40–2.77)	0.90
Psoriasis arthritis	1.55 (0.76–3.16)	–	
AST > 32 U/L	5.45 (1.73–17.1)	9.27 (1.15–74.3)	0.036
ALT > 33 UL	3.67 (1,80–7.43)	0.99 (0.31–3.18)	> 0.99
GGT > 40 U/L	2.85 (1.48–5.48)	0.91(0.33–2.48)	0.85
Cholesterol > 200 mg/dL	1.56 (0.85–2.81)	–	
LDL-cholesterol > 100 mg/dL	2.26 (1.11–4.59)	1.12 (0.85–3.57)	0.46
Triglycerides > 150 mg/dL	4.07 (2.16–7.67)	2.98 (1.13–7.87)	0.026
Albumin < 3.5 g/dL	0.67 (0.06–7.52)	–	
Hemoglobin < 11.5 g/dL	NA	–	
White blood cells < 4.5 × 10^9^/L	0.26 (0.03–2.30)	–	
Platelets < 150 × 10^9^/L	0.21 (0.02–1.84)	–	
ERS ≥ 16 mm	1.45 (0.81–2.60)	–	
Hs-CRP ≥ 0.1 mg/mL	3.95 (1.85–8.43)	2.58 (0.86–7.76)	0.088
Non-viable BT	2.65 (1.34–5.24)	2.14 (0.75–5.95)	0.16

*AST* aspartate aminotransferase, *ALT* alanine aminotransferase, *BMI* body mass index, *BT* bacterial translocation, *CI* confidence interval, *ERS* erythrocyte sedimentation rate, *GGT* g-glutamyl transferase, *hs-CPR* high-sensitivity C-reactive-protein, *HOMA-IR* homeostatic model assessment of insulin resistance, *LDL* low-density lipoprotein, *NA* not available, *OR* odds ratio.

*Abnormal waist circumference: women, ≥ 86 cm, ≥ 95 cm male.

^†^HOMA-IR available in 211 patients.

Comparing mild and moderate-to-severe NAFLD cases, significant differences were found in age; high BMI; prevalence of smoking, metabolic syndrome, and non-viable BT; and abnormal AST and ALT (Supplementary Table S2).

### Correlation of clinical severity scales of psoriasis and NAFLD

Patients with both psoriasis and NAFLD presented higher absolute psoriasis area severity index (PASI), body surface area (BSA), and physician’s global assessment (PGA) (p < 0.01) than those with psoriasis alone. These groups were similar in terms of psoriasis symptoms and quality of life (Table 2).

### Profile of cytokines and NAFLD

**Figure 1:**
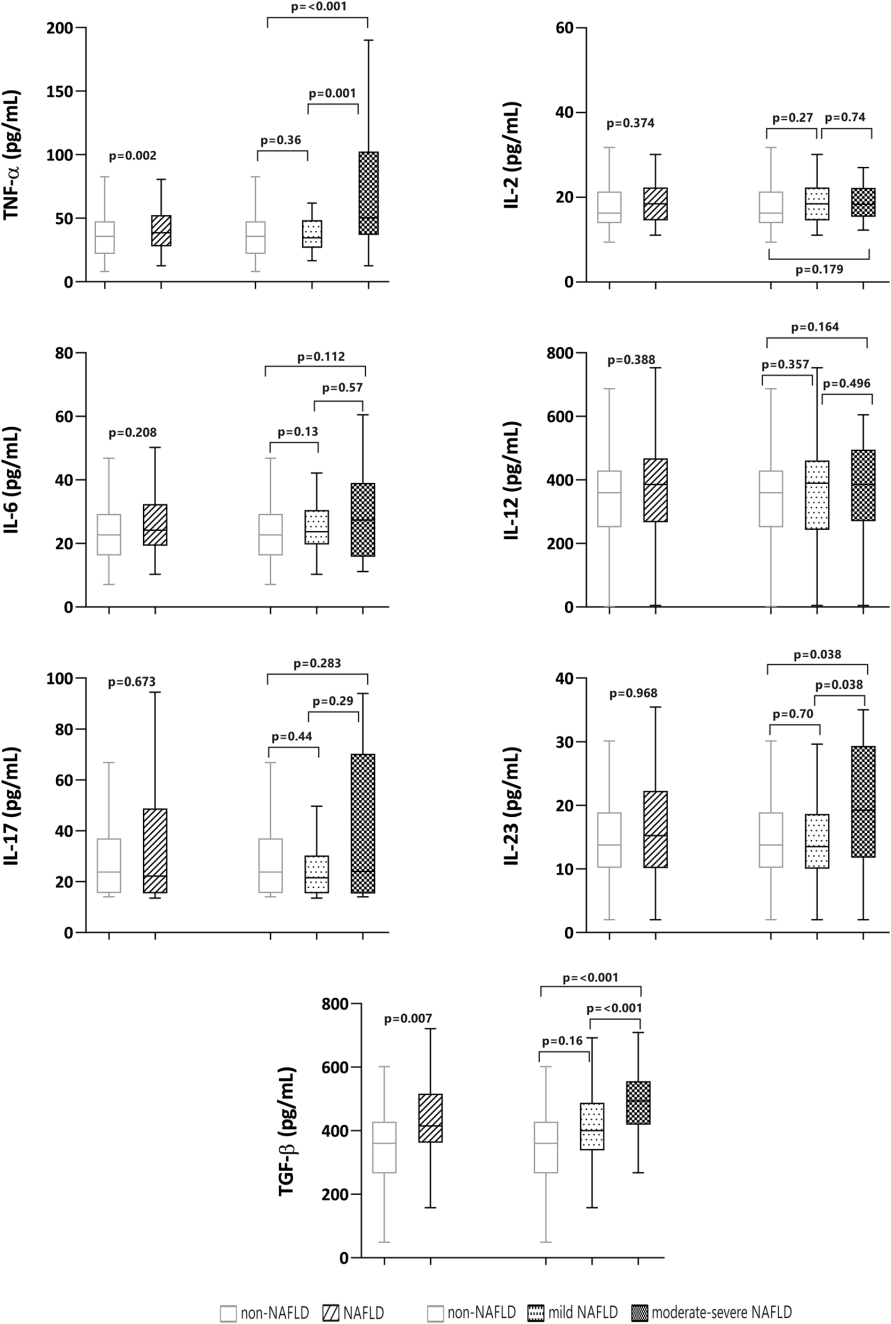
Cytokine values in participants with and without non-alcoholic fatty liver disease (NAFLD).

**Figure 2:**
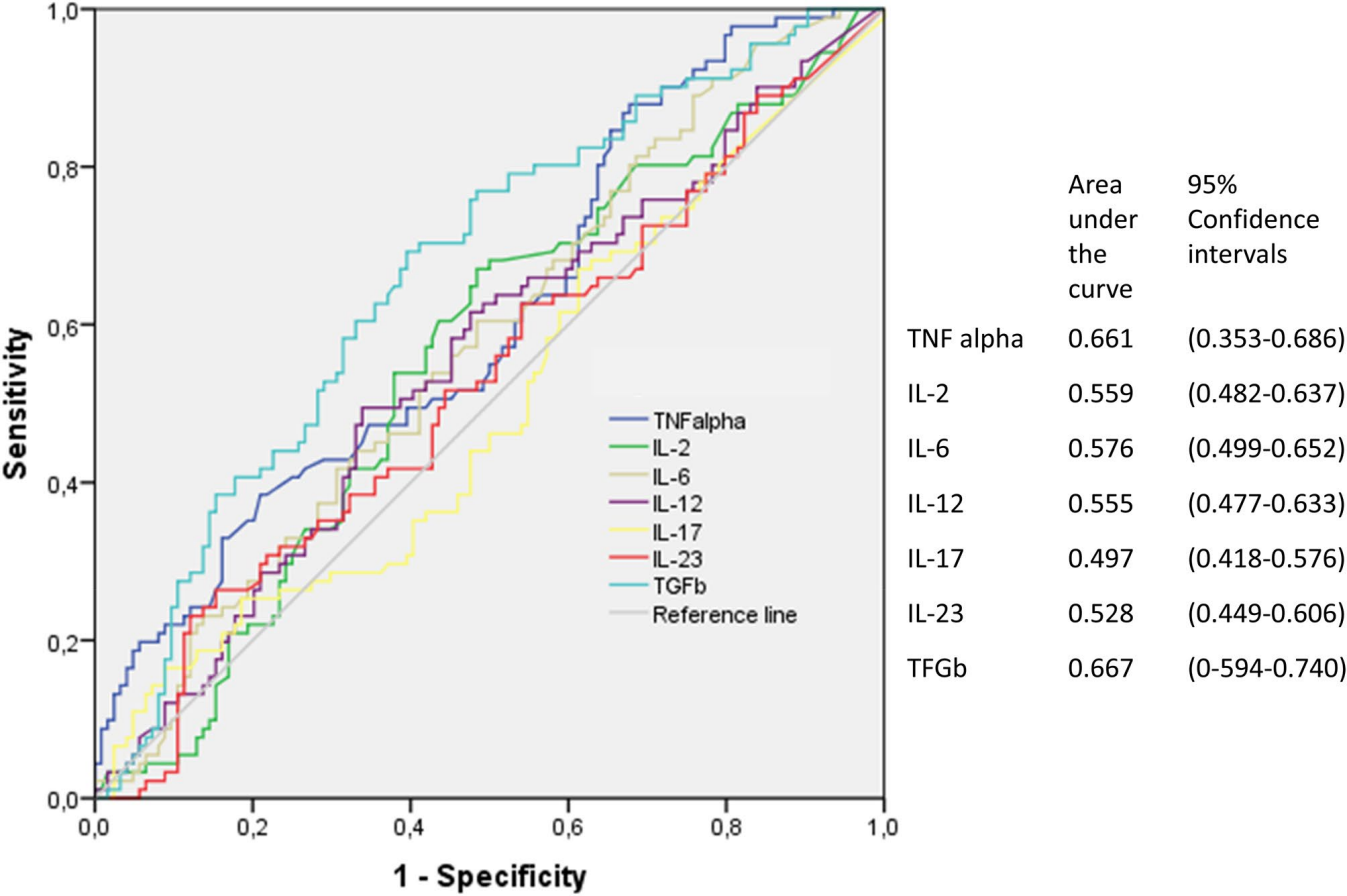
Sensitivity and (1 − specificity) plot and area under the receiver operating characteristic curve for cytokines and non-alcoholic fatty liver disease (NAFLD).

Psoriatic patients with NAFLD had significantly higher levels of TNF-α (p = 0.002), transforming growth factor-beta (TGF-β) (p = 0.007), and IL-23 (p < 0.001) than those without NAFLD (Fig. 1). In the receiver operating characteristic (ROC) analysis, only TNF-α and TGF-β reached statistical significance for the discrimination of NAFLD, with area under curve (AUC) values of 0.64 and 0.68, respectively, using the following cutoffs: TNF-α, 25.48 pg/mL and TGF-β, 382.6 pg/mL (Fig. 2). Sensitivity and specificity are shown in Supplementary Table S3.

In an exploratory multivariable analysis, testing associations with NAFLD, we introduced TNF-α, TGF-β, age, sex, HOMA-IR ≥ 2.15, and BMI ≥ 30 kg/m^2^ into the logistic regression model. We observed independent associations between NAFLD and age (adjusted odds ratio [OR] 1.04; 95% coefficient interval [CI] 1.01–1.05; p = 0.005), male sex (adjusted OR 2.79; 95% CI 1.34–5,83; p = 0.006), values of TNF-α ≥ 20.6 pg/mL (adjusted OR 3.11; 95% CI 1.29–7.63; p = 0.011); BMI ≥ 30 kg/m^2^ (adjusted OR 3.63; 95% CI 1.76–7.49; pa0.001; and HOMA-IR ≥ 2.15 (adjusted OR 6.49; 95% CI 2.56–16.43; p < 0.001). In this model, the p value for the Hosmer–Lemeshow goodness-of-fit test was 0.75, and the AUC of 0.79 indicated good predictive ability.

### Profile of cytokines and non-viable BT

**Figure 3:**
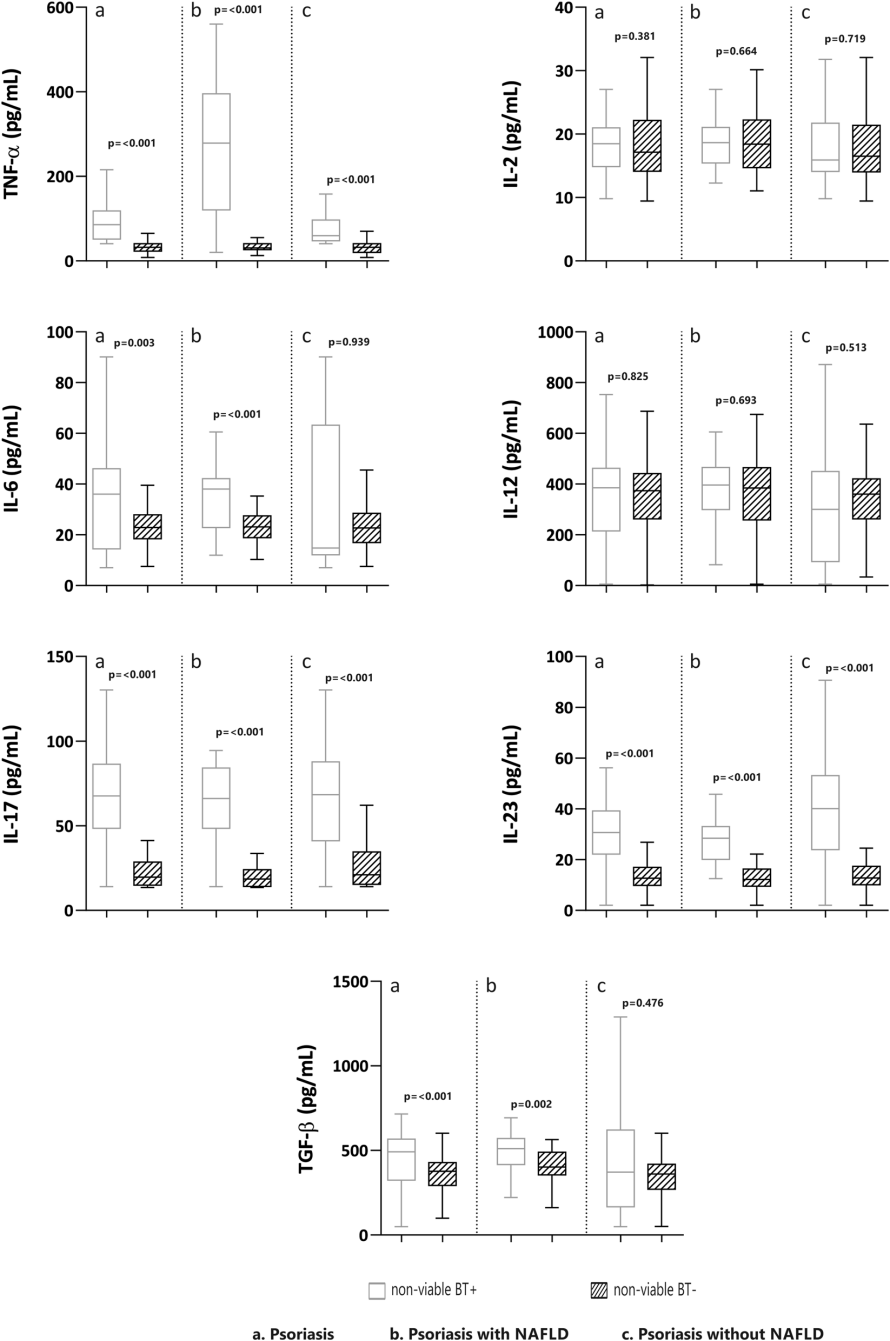
Cytokine values in participants with and without non-alcoholic fatty liver disease (NAFLD), according to bacterial translocation (BT).

Among the 215 patients studied, 44 had non-viable BT (20.6%). These patients had significantly (p < 0.01) higher levels of TNF-α, IL-6, IL-17, IL-23 and TGF-β than those without BT, regardless of whether or not they had NAFLD. However, BT was more prevalent in patients with NAFLD (29.7% vs 13.7%; p < 0.001), who had higher values of TNF-α, Il-6, IL-17, IL-23, and TGF-β than those with NAFLD but without BT (p < 0.001). In contrast, patients without NAFLD and with BT only had significantly elevated TNF-α, IL-17, and IL-23 compared to those without BT (p < 0.001) (Fig. 3).

### NASH and liver fibrosis in psoriatic patients with NAFLD

Liver stiffness measurement (LSM) by transient elastography was performed in 87 of the 91 patients with NAFLD. Fifteen (17.2%) had an LSM of more than 7.8 kPa, suggesting a high risk of liver fibrosis; 13 of these patients underwent liver biopsy to assess liver fibrosis and NASH (Table 3). NASH was diagnosed in only one. Five of the 87 (5.7%) patients with NAFLD had liver fibrosis confirmed by histology; however, only one patient had advanced liver fibrosis (F3).

**Table 3:** Results of liver biopsy in 13 patients with liver stiffness measurement > 7.8 kPa by transient elastography.

Patient	Age (year)	Gender	Fibroscan (KPa)	FIB-4	NFS	Liver biopsy findings					
						Steatosis	Lobular inflammation	Ballooning	Fibrosis	NAS	NASH
1	57	Woman	7.8	0.89	− 1.38	2	1	1	F2	4	Yes
2	53	Man	7.9	0.98	− 0.64	2	1	0	F0	3	Borderline
3	42	Man	8.1	0.62	− 1.29	2	0	0	F0	2	No
4	56	Woman	8.3	1.5	0.9	2	0	0	F0	2	No
5	34	Man	8.9	0.3	− 1617	0	0	0	F0	0	No
6	48	Man	9.7	1.1	− 1.48	2	1	0	F1	3	Borderline
7	51	Woman	10.1	0.62	− 1.14	1	0	0	F0	1	No
8	45	Man	10.40	0.62	− 1.87	3	0	0	F0	3	Borderline
9	55	Man	10.60	0.95	2.03	2	0	0	F1	2	No
10	66	Woman	10.80	1.38	0.23	2	0	0	F3	2	No
11	60	Woman	11	0.83	− 1.18	3	1	0	F0	4	Borderline
12	63	Man	13.9	0.83	− 0.83	2	1	0	F0	3	Borderline
13	62	Woman	16.8	1.97	0.066	3	0	0	F1	3	Borderline

FIB-4 is a non-alcoholic fatty liver disease (NAFLD) fibrosis score: FIB-4 < 1.3 Low Risk of Liver Fibrosis, FIB-4 130–2.26: Indeterminate Risk of Liver Fibrosis "grey area", FIB-4 > 3.26: High Risk of Liver Fibrosis; NFS: NAFLD fibrosis score: NFS < − 1.455: Low Risk of Liver Fibrosis, NFS − 1.455–0.672: Indeterminate Risk of Liver Fibrosis "grey area" NFS > 0.672 : High Risk of Liver Fibrosis; NAS: NAFLD activity score; NASH: non-alcoholic steatohepatitis.

A higher proportion of patients with NAFLD and a high risk of fibrosis according to LSM had diabetes mellitus, BMI over 30 kg/m^2^, ALT over 33 UI, and ERS over 16 mm, compared to patients with a low risk of fibrosis (Supplementary Table S4). Multivariable logistic regression analysis confirmed this association for diabetes mellitus and high ALT and ERS (Supplementary Table S5). Patients with high and low LSM had a similar cytokines profile (Supplementary Table S6).

## Discussion

This study shows a prevalence of NAFLD of 42.3% in psoriatic patients—higher than the prevalence reported in the general population in Spain (25.8% to 33.4% in men and 20.3% in women)^14,23^, and consistent with other prevalence studies in people with psoriasis (44% to 65.6%)^6,7,15,24,25^.

The pathogenesis of psoriasis comorbidities, including NAFLD, is not completely understood. Different factors are known to be involved, including common patterns of immune responses and inflammatory pathways, genetic predisposition, and shared risk factors^5^. These risk factors are largely similar to those in the general population and are related to metabolic syndrome^26,27^.

In our patients, NAFLD was independently related to male sex, age, and metabolic syndrome comorbidities. These results are in line with previous studies linking metabolic syndrome with NAFLD^28,29^, which could be related to insulin resistance as a shared pathogenic pathway in both NAFLD and metabolic syndrome in people with psoriasis.

In keeping with previous reports^7,25,27,30^, our patients with NAFLD had a greater PASI compared to those without. However, severity scores in our patients were generally low because most were under systemic therapy, so caution is warranted when interpreting this result. When analyzing specific psoriasis treatments, only acitretin appeared to be associated with NAFLD; however, other studies have not detected any relationship^25,31,32^.

Patients on methotrexate did not show an increased risk of liver steatosis, in agreement with previous studies^27,33^. This finding is notable since methotrexate is frequently used for treating psoriasis but is involved in the development of liver fibrosis^34^, especially when used long-term in patients with other risk factors for fatty liver disease, like excessive alcohol use, obesity, and diabetes^35^. However, in single dose regimens of methotrexate 5 mg to 15 mg weekly, plus folate supplementation, fibrosis and clinically apparent liver disease are rare even with long-term use, as observed in our study^36^.

According to ultrasound-based grading of NAFLD severity, participants with moderate-to-severe NAFLD were older and more obese, and they showed more pronounced abnormalities in their metabolic profile, in line with other studies^37^. Moreover, these patients had a higher level of transaminases and non-viable BT, probably related to increased shared inflammatory status, which in turn could be linked to psoriasis activity at the time of the study.

Patients with NAFLD also presented greater inflammatory activity, as determined by the cytokines TNF-α and TGF-β, and this activity was greater in patients with moderate-to-severe NAFLD. Pro-inflammatory cytokines, including TNF-α, play a crucial role in pathogenesis of both NAFLD and psoriasis, as well as in the progression of NAFLD to non-alcoholic steatohepatitis (NASH). Indeed, psoriasis-related inflammation could trigger the development of NAFLD^2^.

Previous studies have shown that bacterial DNA can translocate to extra-intestinal sites and promote an immunological response similar to that produced by viable bacteria, giving rise to an inflammatory state that can complicate chronic liver disease^38,39^. Mechanisms proposed to promote BT include increased gut permeability, intestinal bacterial overgrowth, and alterations in the gut microbiota composition^40–43^. This is added a with genetic predisposition to BT and inflammation as seen in other clinical situation as Crohn's disease^33,44^. So a new line of research is opened on alterations in genes related to the inflammatory response as an additional pathogenic mechanism in psoriasis BactDNA in the bloodstream that promotes an immunological response, favoring the release of pro-inflammatory cytokines and perpetuating chronic inflammation^42^. Furthermore, the synthesis of cytokines, mainly TNF-α, increases intestinal permeability, probably bringing on BT from the intestinal lumen to the bloodstream^45^, and it has been associated with inflammatory conditions, such as inflammatory bowel disease^42^, and maybe psoriasis. Ramirez et al.^46^ suggest a role for BT in active plaque psoriasis, which is more evident in patients with longer duration and earlier onset of the disease. Additionally, our results indicate that BT is more frequent in psoriatic patients with versus without NAFLD. This finding may be of interest since one of the factors in the development and progression of NAFLD is gut permeability, which may be mediated by the microbiome.

Although the role of the BT-measured microbiome in patients with psoriasis and NAFLD has not been evaluated so far, in our study BT was associated with a higher estimated inflammatory response (elevation of proinflammatory cytokines TNF-α and TGF-β) in patients with versus without NAFLD.

While patients with both psoriasis and NAFLD have an increased inflammatory response, liver damage, as estimated by the degree of liver fibrosis, was modest in our cohort. Only 5.7% of patients in the total cohort had liver fibrosis on liver biopsy, and only one patient met histological criteria for the diagnosis of NASH. To our knowledge, ours is one of the few published studies that have assessed NASH and liver fibrosis by liver biopsy in patients with psoriasis. In another, the prevalence of NASH in psoriatic patients was about 22%^24^. The low rate of NASH in our series may be partly due to the fact that 68% of NAFLD patients were under systemic treatment, which could modulate the inflammatory response in the liver. Patients at high risk of fibrosis, as estimated by LSM, had a higher prevalence of diabetes, obesity, and elevated transaminases. This association is explained by the important role that both diabetes and metabolic syndrome play in the pathogenesis of liver fibrosis in NAFLD patients^47^.

This study has the limitations inherent in all single-center studies, and the results cannot be extrapolated to a population outside the Mediterranean region, where exposure to sunshine and dietary habits may differ. Moreover, only a small number of patients with elastography values of over 7.8 kPa underwent liver biopsy, which may explain the absence of any observed relationship between fibrosis and clinical and analytical parameters. Another limitation, related to the real-life study design, is the inclusion of patients with very heterogeneous characteristics (naïve patients, in systemic treatment or not, phototherapy, topical treatment). Thus, the severity of psoriasis, as measured by PASI, BSA, and PGA, was low at the time of the study, which could affect the interpretation of the relationship that disease severity has with comorbidities and inflammatory parameters. However, the heterogenous sample could also be considered a strength, as data were obtained from a large population in routine clinical practice. Another strength of the study is its prospective and protocolized nature, ensuring standardized procedures by the research team.

In conclusion, approximately 40% of our patients with moderate-to-severe psoriasis had NAFLD, and this was more common in older, obese men with more severe psoriasis and metabolic syndrome. Psoriatic patients with NAFLD—especially older, obese men with higher HOMA-IR and moderate-to-severe NAFLD—were more likely to have non-viable BT and higher levels of TNF-α and TGF-β. Up to 10% of NAFLD patients showed LSM suggesting a high risk of liver fibrosis, but this condition was confirmed by histology in just 5.7% of the patients. Diabetes mellitus, elevated ALT and ERS were more common in patients at high risk of fibrosis.

Our study suggests that chronic inflammation, represented by higher levels of TNF-α and TGF-β, and non-viable BT could contribute to the development of NAFLD in patients with psoriasis. However, our data are descriptive and do not provide mechanistic evidence to definitively demonstrate this. Additional experimental studies are needed to evaluate the underlying biological mechanisms and confirm this pathogenic association.

## Material and methods

### Design and setting

This prospective, descriptive observational study took place at the psoriasis unit of the dermatology service in the General University Hospital of Alicante, located on the south-eastern Mediterranean coast of Spain. This unit serves a population of approximately 267,000 people.

### Participants and data collection

Inclusion criteria were: aged at least 18 years, with a diagnosis of moderate-to-severe psoriasis^48^, , presenting to our center from 1 September 2017 to 31 May 2018, and providing informed consent. Exclusion criteria along with epidemiological, clinical, and laboratory variables are recorded in Supplementary Table S7. Metabolic syndrome was defined according to the National Cholesterol Education Program Adult Treatment Panel III^49^. Analytical parameters were dichotomized according to the thresholds for normal values used in our laboratory^50^.

Active treatment was defined as that which started at least six months before, and was classified into five categories: topical treatment, phototherapy, systemic treatment (methotrexate, retinoids, and cyclosporine), apremilast and biologics (including both anti-TNF, anti-IL-12/23, anti-IL-17, and anti-IL-23 therapies). Severity was determined according to the BSA affected, PASI, and PGA (range 0 to 4). Visual analog scales were employed to measure pain and itch related to psoriasis, and the DLQI to assess quality of life.

### Identification of non-viable bacterial translocation

Non-viable BT was defined as the presence of bactDNA in blood in a negative microbiological culture. Total DNA extraction from homogenized specimens was undertaken with the QIAamp DNA Tissue kit (QIAgen, Barcelona, Spain). A standard PCR followed by partial nucleotide sequencing of the 16SrRNA gene was performed according to the methodology described elsewhere^51^. Two microliters of template were added into a reaction mix containing 10 mmol/L Tris buffer (pH 8.3), 50 mmol/L KCl, 1.5 mmol/L Mg_2_, 200 μmol/L of each deoxynucleoside triphosphate, 50 pmol of primers 5′-AGAGTTTGAT-CATGGCTCAG-3′ and 5′-ACCGCGACTGCTGCT-GGCAC-3′, and 1.25 U BioTaq (Bioline, London, England) to reach a final volume of 50 μL. The primers located at positions 7–27 and 531–514 (*Escherichia coli* numbering) are universal eubacterial primers that will amplify any known bacterial 16S ribosomal RNA gene. A 35-cycle PCR was run in a GeneAmp 9700 (Applied Bio-systems, Foster City, CA) at: 94 °C for 30 s, 55 °C for 30 s, and 72°. The detection limit of the technique was 5 pg/mL of bacterial DNA. Samples under detection limit were considered negative.

### Measure of serum cytokine levels

Enzyme-linked immunoabsorbent assays (ELISAs) were performed in serum for TNF-α, IL-2, IL-6, IL-17, IL-23, IL-12 and (TGF-β (Rat Quantikine kits, R&D Systems, Minneapolis, MN, USA). All samples were tested in triplicate and read in a Sunrise Microplate Reader (Tecan, Männedorf, Switzerland). The lower limit of detection for each assay was 5 pg/mL. Standard curves were generated for every plate, and the average zero standard optical densities were subtracted from the rest of the standards, controls, and samples to obtain a corrected concentration.

### Assessment of NAFLD

A board-certified radiologist performed hepatic ultrasound in all participants, using a Toshiba Aplio 300 and Aplio 500 ultrasound scanner with a 3.5-MHz Convex abdominal probe. All patients included underwent a hepatic ultrasound to assess hepatic steatosis, which was defined by unique features including bright hepatic echoes, increased hepatorenal echogenicity, and vascular blurring of the portal or hepatic vein. Severity was defined as grade 1 (mild steatosis), 2 (moderate), or 3 (severe steatosis), as described elsewhere^52^.

NAFLD was diagnosed in patients with hepatic steatosis after excluding secondary causes of steatosis. We classified NAFLD patients in two groups based on the severity of steatosis, as assessed by ultrasound: mild NAFLD and moderate-to-severe NAFLD.

Non-invasive tests for liver fibrosis were performed in all NAFLD patients: NAFLD fibrosis score (NFS), Fibrosis-4 (FIB-4), and hepatic transient elastography by Fibroscan, as described elsewhere^53^. Patients with LSM of more than 7.8 kPa underwent liver biopsy to confirm advanced liver fibrosis and/or NASH. Liver biopsies were performed percutaneously with ultrasound guidance and 16 G × 15 cm tru-cut needles (Biopince Full core Biopsy Instrument; Argon medical Devices, TX USA). Liver histology was classified according to the NAFLD activity score (NAS)^54^. NASH was defined as steatosis plus lobular inflammation and ballooning degeneration^55^.

### Statistical analysis

Descriptive statistics were used to summarize the data. Categorical variables were compared according to the presence of NAFLD and explanatory variables including age, sex, and metabolic syndrome, using the chi-squared test of homogeneity. We undertook a ROC curve analysis, establishing a cutoff for each of the cytokines to identify individuals who had NAFLD.

Crude ORs and 95% CIs were calculated to assess the association between explanatory variables and both NAFLD and risk of fibrosis. All variables yielding a p value of less than 0.05 were included in a saturated, multivariable logistic regression model. All analyses were carried out IBM SPSS Statistics v25 for Mac (Armonk, NY) or Prism (GraphPad).

### Ethical aspects

The institutional review board of University General Hospital of Alicante & Alicante Institute of Sanitary and Biomedical Research (ISABIAL), reviewed and approved the study protocol (Ref. CEIC PI2017/27). All participants provided written informed consent. Data confidentiality and patient anonymity were maintained at all times, in accordance with Spanish regulations on observational studies^56^.

## Supplementary Information

Supplementary Information.

## Abbreviations

ALT: Alanine aminotransferase
AST: Aspartate aminotransferase
AUC: Area under curve
BAS: Body surface area
BMI: Body mass index
BT: Bacterial translocation
DLQI: Dermatology life quality index
ELISA: Enzyme-linked immunoabsorbent assays
ESR: Erythrocyte sedimentation rate
GGT: Gamma-glutamyl transferase
FIB-4: Fibrosis-4
HOMA-IR: Homeostatic model assessment of insulin resistance
hs-CRP: High-sensitivity C-reactive protein
IFN: Interferon
IL: Interleukin
LSM: Liver stiffness measurement
NAFLD: Non-alcoholic fatty liver disease
NAS: NAFLD activity score
NASH: Non-alcoholic steatohepatitis
NFS: NAFLD fibrosis score
OD: Odds ratio
PASI: Psoriasis area severity index
PCR: Polymerase chain reaction
PGA: Physician’s global assessment
ROC: Receiver operating characteristic
TGF: Transforming growth factor
TNF: Tumor necrosis factor
