# Supplementary material for: Non-alcoholic fatty liver disease is associated with bacterial translocation and a higher inflammation response in psoriatic patients
Source: Sci Rep. 2021 Apr 21;11:8593. doi: 10.1038/s41598-021-88043-8 (PMC8060289; doi:10.1038/s41598-021-88043-8)
Supplement: Supplementary file 1 — Supplementary Information. [file 41598_2021_88043_MOESM1_ESM.docx]

**Supplementary material**

**Title:** Non-alcoholic fatty liver disease is associated with bacterial translocation and a higher inflammation response in psoriatic patients

**Authors:**

Isabel Belinchón-Romero, Pablo Bellot, David Romero-Pérez, Isolina Herraiz-Romero, Francisco Marco, Rubén Frances, José-Manuel Ramos-Rincón

1. Dermatology Department, University General Hospital of Alicante & Alicante Institute of Sanitary and Biomedical Research (ISABIAL), Alicante, Spain

2. Clinical Medicine Department, Miguel Hernández University of Elche, Spain

3. Digestive Medicine Department, University General Hospital of Alicante & Alicante Institute of Sanitary and Biomedical Research (ISABIAL), Alicante, Spain

4. Dermatology Unit, Hospital Quirón-Salud, Tenerife, Spain

5. Radiodiagnostic Department, University General Hospital of Alicante & Alicante Institute of Sanitary and Biomedical Research (ISABIAL), Alicante, Spain

6. Immunology Department, University General Hospital of Alicante & Alicante Institute of Sanitary and Biomedical Research (ISABIAL), Alicante, Spain

7. Research Institute, University General Hospital of Alicante & Alicante Institute of Sanitary and Biomedical Research (ISABIAL), Alicante, Spain

8. CIBERehd, Instituto de Salud Carlos III, Madrid, Spain.

9. Internal Medicine Department, University General Hospital of Alicante-ISABIAL, Spain

**Authors, orcid, emails**

| Isabel Belinchón-Romero | <http://orcid.org/0000-0002-6007-7320> | belinchon_isa@gva.es |
| --- | --- | --- |
| Pablo Bellot | https://orcid.org/0000-0001-8307-1813 | pablobellot.garcia@gmail.com |
| David Romero-Pérez | https://orcid.org/0000-0003-1875-6722 | davidromero66@hotmail.com |
| Isolina Herraiz-Romero | [http://orcid.org/0000-0002-6007-7320](http://orcid.org/0000-0002-6007-7320?lang=en) | i[solinaherraizromero@gmail.com](mailto:solinaherraizromero@gmail.com) |
| Francisco Marco | https://orcid.org/0000-0001-5113-3637 | marco_fradela@gva.es |
| Rubén Frances | https://orcid.org/0000-0001-5105-1201 | rfrances@umh.es |
| Jose Manuel Ramos Rincón | https://orcid.org/0000-0002-6501-9867 | jose.ramosr@umh.es |

**Supplementary Figure S1.** Flow chart

**
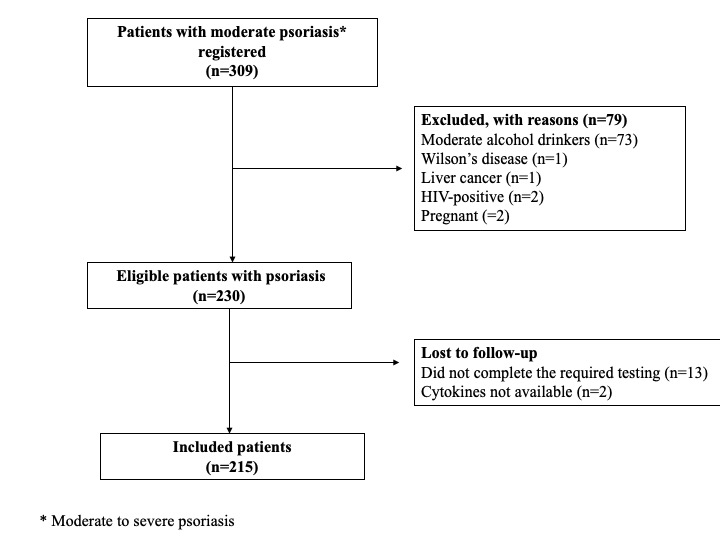
**

**Supplementary Table S1.** Treatments for psoriasis in participants with and without non-alcoholic fatty liver disease (NAFLD)

| **Variable** | **No NAFLD**  **(n=124)** | **NAFLD**  **(n=91)** | **P value** |
| --- | --- | --- | --- |
| Patients without treatment | 81 (65.3) | 62 (68.1) | 0.72 |
| Patients with treatment | 43 (34.7) | 29 (31.8) | 0.72 |
| Non-systemic treatment |  |  |  |
| Topical treatment | 30 (24.2) | 16 (17.6) | 0.24 |
| Narrowband UVB phototherapy | 14 (11.3) | 13 (14.3) | 0.21 |
| Systemic treatment |  |  |  |
| Adalimumab | 10 (8.1) | 6 (6.6) | 0.68 |
| Etanercept | 8 (6.5) | 7 (7.7) | 0.72 |
| Golimumab | 1 (0.8) | 0 | 0.99 |
| Guselkumab | 1 (0.8) | 0 | 0.39 |
| Infliximab | 2 (1.6) | 1 (1.1) | 0.99 |
| Secukinumab | 22 (17.7) | 13 (14.3) | 0.49 |
| Ustekinumab | 17 (13.7) | 17 (18.7) | 0.35 |
| Acitretin | 4 (3.2) | 10 (11.0) | 0.023 |
| Apremilast | 2 (1.6) | 2 (2.2) | 0.99 |
| Methotrexate | 14 (13.3) | 7 (7.7) | 0.38 |
| Isotretinoin | 0 | 1 (1.1) | 0.24 |
| Leflunomide | 0 | 1 (1.1) | 0.43 |

**Supplementary Table S2.** Epidemiological, clinical, and laboratory characteristics of psoriatic patients without non-alcoholic fatty liver disease (NAFLD), with mild NAFLD, and with moderate-severe NAFLD

|  | **No NAFLD (n=124)** | **Mild NAFLD (n=62)** | **Moderate-severe NAFLD (n=29)** | **P value** | | |
| --- | --- | --- | --- | --- | --- | --- |
|  |  |  |  | **A v B** | **A vs C** | **B vs C** |
| Men, n (%) | 61 (49.2%) | 40 (64.5) | 19 (65.6) | 0.048 | 0.11 | 0.99 |
| Age in years, median (IQR) | 41.5 (35-51) | 53 (42-61) | 56 (48-62) | <0.001 | <0.001 | 0.05 |
| Years of disease evolution, median (IQR) | 16 (10-23.5) | 18.5 (9-28) | 25 (11.5-30) | 0.55 | 0.073 | 0.22 |
| BMI ≥ 30 kg/m^2^, n (%) | 26 (21.0) | 31 (50.0) | 23 (79.3) | <0.001 | <0.001 | 0.008 |
| Abnormal waist circumference*, n (%) | 71 (57.3) | 54 (87.1) | 28 (96.6) | <0.001 | <0.001 | 0.26 |
| Smokers, n (%) | 50 (40.3) | 20 (32.3) | 3 (10.3) | 0.285 | 0.002 | 0.021 |
| HOMA-IR ≥2.15^†^, n (%) | 57 (47.5) | 55 (88.7) | 28 (96.6) | <0.001 | <0.001 | 0.428 |
| Diabetes mellitus, n (%) | 8 (6.5) | 13 (21.0) | 10 (34.5) | 0.003 | <0.001 | 0.17 |
| Cardiopathy, n (%) | 5 (4.0) | 6 (9.7) | 4 (13.8) | 0.12 | 0.067 | 0.55 |
| Dyslipidemia, n (%) | 45 (36.3) | 39 (62.9) | 18 (62.1) | 0.001 | 0.011 | 0.99 |
| Metabolic syndrome, n (%) | 30 (24.2) | 32 (51.6) | 22 (75.9) | <0.001 | <0.001 | 0.028 |
| Psoriasis arthritis, n (%) | 18 (14.5) | 16 (25.8) | 3 (10.3) | 0.06 | 0.55 | 0.09 |
| AST >32 U/L, n (%) | 4 (3.2) | 5 (8.1) | 9 (31.0) | 0.15 | <0.001 | 0.01 |
| ALT >33 UL, n (%) | 14 (11.3) | 12 (12.4) | 17 (58.6) | 0.135 | <0.001 | <0.001 |
| GGT > 40 U/L, n (%) | 19 (15.3) | 18 (29.0) | 13 (44.8) | 0.027 | <0.001 | 0.16 |
| Cholesterol > 200 mg/dL, n (%) | 33 (26.8) | 23 (37.1) | 10 (34.5) | 0.14 | 0.396 | 0.99 |
| LDL-cholesterol >100 mg/dL, n (%) | 90 (72.6) | 52 (83.9) | 26 (89.7) | 0.088 | 0.053 | 0.53 |
| Triglycerides > 150 mg/dL, n (%) | 20 (16.1) | 25 (40.3) | 15 (51.7) | <0.001 | <0.001 | 0.30 |
| Albumin < 3.5 g/dL, n (%) | 2 (1,6) | 1 (1.6) | 0 (0) | 0.99 | 0.99 | 0.99 |
| Hemoglobin <11.5 g/dL, n (%) | 0 (0.0) | 0 (0,0) | 0 (0) | 0.99 | 0.99 | - |
| White blood cells < 4.5× 10^9^/L, n (%) | 5 (4.0) | 0 (0.0) | 1 (3.4) | 0.17 | 0.99 | 0.31 |
| Platelets < 150 × 10 ^9^ /L, n (%) | 6 (4.8) | 1 (1.6) | 0 (0.0) | 0.27 | 0.59 | 0.99 |
| ERS > 16 mm, n (%) | 34 (28.1) | 26 (41.9) | 7 (24.1) | 0.059 | 0.66 | 0.10 |
| Hs-CRP ≥ 0.1mg/mL, n (%) | 82 (67.2) | 53 (85.5) | 28 (96.6) | 0.008 | 0.001 | 0.11 |
| Non-viable BT, n (%) | 17 (13.7) | 12 (19.4) | 14 (48.3) | 0.31 | <0.001 | 0.002 |

*Abnormal waist circumference: ≥86 cm in women or ≥ 95 cm in men

^†^HOMA-IR available in 211 patients

AST: aspartate aminotransferase, ALT: alanine aminotransferase, BMI: body mass index, BT: bacterial translocation, ERS: erythrocyte sedimentation rate, GGT: g-glutamyl transferase, IQR: interquartile range, hs-CPR: high-sensitivity C-reactive-protein, HOMA-IR: homeostatic model assessment of insulin resistance, LDL: low-density lipoprotein.

**Supplementary Table S3**. Cytokines cutoff values for discriminating non-alcoholic fatty liver disease (NAFLD)

| **Cytokine** | **Cutoff (pg/mL)** | **P-value** | **Sensitivity** | **Specificity** |
| --- | --- | --- | --- | --- |
| TNF-α | 25.48 | 0.008 | 0.88 | 0.33 |
| IL-2 | 16.42 | 0.14 | 0.67 | 0.50 |
| IL-6 | 15.65 | 0.06 | 0.89 | 0.23 |
| IL-12 | 392.50 | 0.17 | 0.49 | 0.65 |
| IL-17 | 62.98 | 0.93 | 0.16 | 0.90 |
| IL-23 | 20.60 | 0.48 | 0.25 | 0.80 |
| TGF-β | 382.60 | < 0.001 | 0.69 | 0.60 |

**Supplementary Table S4.** Epidemiological, clinical, and laboratory characteristics in participants with high versus low liver stiffness measurement (LSM)

| **Variable** | **High LSM***  **(n= 15)** | **Low LSM***  **(n=72)** | **P value** |
| --- | --- | --- | --- |
| Male, n (%) | 8 (53.3) | 46 (66.7) | 0.4 |
| Age in years, median (IQR) | 53 (45-60) | 53.5 (45.3-61.8) | 0.96 |
| BMI ≥30 kg/m^2^, n (%) | 15 (100) | 37 (51) | 0.005 |
| Abnormal waist circumference*, n (%) | 15 (100) | 63 (88) | 0.14 |
| Smokers, n (%) | 1 (6.6) | 20 (27) | 0.07 |
| HOMA-IR ≥2.15, n (%) | 15 (100) | 64 (81) | 0.17 |
| Diabetes mellitus, n (%) | 7 (46.7) | 16 (22.2) | 0.05 |
| Cardiopathy, n (%) | 2 (13) | 6 (8.3) | 0.41 |
| Dyslipidemia, n (%) | 12 (80) | 39 (56) | 0.07 |
| Metabolic syndrome, n (%) | 11 (73) | 41 (56.9) | 0.18 |
| Psoriatic arthritis, n (%) | 2 (13) | 17 (23.6) | 0.31 |
| AST >32 U/L, n (%) | 5 (33.3) | 10 (13) | 0.06 |
| ALT >33 UL, n (%) | 12 (80) | 22 (30.5) | <0.001 |
| GGT > 40 U/L, n (%) | 8 (53) | 24 (33) | 0.14 |
| Cholesterol >200 mg/dL, n (%) | 7 (46) | 26 (36.1) | 0.44 |
| LDL-cholesterol >100 mg/dL, n (%) | 11 (73.3) | 64 (88.8) | 0.11 |
| Triglycerides >150 mg/dL, n (%) | 9 (60) | 28 (38.8) | 0.3 |
| Albumin <3.5 g/dL, n (%) | 1 (6.6) | 0 (0.0) | 0. |
| Hemoglobin <11.5 g/dL, n (%) | 0 (0) | 0 (0) | -- |
| White blood cells < 4.5× 10^9^/L, n (%) | 1 (6.6) | 0 (0) | 0.02 |
| Platelets <150 × 10 ^9^ /L, n (%) | 0 (0) | 2 (2.7) | 0.5 |
| ERS ≥16 mm, n (%) | 11 (73) | 25 (34.7) | 0.005 |
| Hs-CRP ≥ 0.1mg/mL n (%) | 14 (93.7) | 62 (86.1) | 0.39 |
| Non-viable BT, n (%) | 5 (33.3) | 19 (27.5) | 0.45 |

* High liver stiffness measurement (LSM) was defined as LSM ≥ 7.8 KPa , Low LSM was defined as LSM < 7.8 KPa

^†^Abnormal waist circumference: ≥86 cm in women or ≥ 95 cm in men

AST: aspartate aminotransferase, ALT: alanine aminotransferase, BMI: body mass index, BT: bacterial translocation, ERS: erythrocyte sedimentation rate, GGT: g-glutamyl transferase, IQR: interquartile range, hs-CPR: high-sensitivity C-reactive-protein, HOMA-IR: homeostatic model assessment of insulin resistance, LDL: low-density lipoprotein.

**Supplementary Table S5.** Risk of developing high liver stiffness measurement (LSM)*, according to explanatory variables (bivariable and multivariable analysis)

| **Variable** | **Crude OR**  **(95% CI)** | **Adjusted OR (95% CI)** | **P value** |
| --- | --- | --- | --- |
| Men | 0.64 (0.21-1.98) | - | - |
| Age | 0.99 (0.94-1.03) | - | - |
| BMI kg/m^2^ ≥ 30 | NC | NC | - |
| Abnormal waist circumference^†^ | NC | NC | - |
| Smoker | NC | NC | - |
| HOMA-IR ≥ 2.15 | NC | NC | - |
| Diabetes mellitus | **3.06 (1.01-9.7)** | **5.24 (1.15-23.7)** | 0.031 |
| Cardiopathy | 1.69 (0.30-9.31) | - | - |
| Dyslipidemia | 3.02 (0.78-11.6) | - | - |
| Metabolic syndrome | 2.07 (0.64-7.15) | - | - |
| Psoriasis arthritis | 0.49 (0.10-2.42) | - | - |
| AST > 32 U/L | 3.1 (0.85-11.0) | - | - |
| ALT > 33 UL | **7.14 (2.03-25)** | **16.49 (3.15-86.35)** | 0.001 |
| GGT > 40 U/L | 2.41 (0.78-7.5) | - | - |
| Cholesterol > 200 mg/dL | 1.25 (0.40-3.92) | - | - |
| LDL-cholesterol > 100 mg/dL | 0.34 (0.8-1.23) | - | - |
| Triglycerides >150 mg/dL | 2.35 (0.75-7.34) | - | - |
| Albumin < 3.5 g/dL | NC | NC | - |
| Hemoglobin < 11.5 g/dL | NC | NC | - |
| White blood cells < 4.5× 10^9^/L | NC | NC | - |
| Platelets < 150 × 10 ^9^ /L | NC | NC | - |
| ERS ≥ 16 mm | **5.14 (1.49-17.91)** | **11.93 (2.30-61.95)** | 0.003 |
| Hs-CRP ≥ 0.1 mg/mL | 2.25 (0.2-19) | - | - |
| Non-viable BT | 1.3 (0.39-4.2) | - | - |

*High liver stiffness measurement (LSM) was defined as LSM ≥ 7.8 KPa, low LSM was defined as LSM < 7.8 KPa

^†^Abnormal waist circumference: ≥86 cm in women or ≥ 95 cm in men

AST: aspartate aminotransferase, ALT: alanine aminotransferase, BMI: body mass index, BT: bacterial translocation, CI: confidence interval, ERS: erythrocyte sedimentation rate, GGT: g-glutamyl transferase, IQR: interquartile range, hs-CPR: high-sensitivity C-reactive-protein, HOMA-IR: homeostatic model assessment of insulin resistance, LDL: low-density lipoprotein, NC: no calculable; OR: odds ratio.

**Supplementary Table S6.** Cytokine values in participants with non-alcoholic fatty liver disease (by liver stiffness measurement (LSM), mean (standard deviation)

,

| **Variable** | **High LSM***  **(n= 15)** | **Low LSM ***  **(n=72)** | **P value** |
| --- | --- | --- | --- |
| TNF-α | 59.3 (39) | 56.03 (45.6) | 0.57 |
| IL-2 | 18.8 (5) | 19.7 (6.5) | 0.9 |
| IL-6 | 25.5 (8.5) | 31.1 (33) | 0.6 |
| IL-12 | 806.7 (1429.4) | 353.6 (210.5) | 0.23 |
| IL-17 | 34.77 (25.9) | 31.7 (23.7) | 0.96 |
| IL-23 | 19.2 (8.9) | 16.1 (9.9) | 0.53 |
| TGF-β | 458.2 (193.5) | 353.6 (210.5) | 0.44 |

* High liver stiffness measurement (LSM) was defined as LSM ≥ 7.8 KPa, low LSM was defined as LSM < 7.8 KPa

**Supplementary Table S7.** Exclusion criteria and dichotomous epidemiological, clinical and analytical variables

| **Exclusion criteria**   - Treatment with systemic corticosteroids in the previous 30 days - Moderate alcohol consumption (> 3 units of alcohol per day in man or more than 2 units in women) - Other chronic liver disease (including B or C hepatitis-infected patients, Wilson’s disease, autoimmune hepatitis, primary biliary cirrhosis, primary sclerosant cholangitis or hepatic malignancy) - Incapacitating disease or cognitive limitation - HIV-infected patients - Pregnancy |
| --- |
| **Dichotomous epidemiological and clinical variables**   - Body mass index (≥ 30 kg/m^2^, < 30 kg/m^2^) - Cardiopathy, including both arrhythmias and ischemic heart disease (yes, no) - Diabetes mellitus (yes, no) - Dyslipidemia (yes, no) - Gender (male, female) - Metabolic syndrome (yes, no) - Smoker (yes, no) - Abnormal waist circumference (yes: ≥ 86 cm in women or ≥ 95 cm in men; no <86 cm in women and < 95 cm in men) |
| **Dichotomous analytical parameters**   - Alanine aminotransferase (>33 U/L, ≤ 33 U/L) - Albumin (<3.5 g/dL, >3.5 g/dL) - Aspartate aminotransferase (>32 U/L, ≤ 32U/L) - Erythrocyte sedimentation rate (≥ 16 mm, < 16 mm) - Gammaglutamyl transferase (> 40 U/L, ≤ 40 U/L) - Glucose (≥ 100 mg/dl, < 100 mg/dl) - Hemoglobin (<11.5 g/dL, ≥ 11.5 g/dL) - High-sensitivity C-reactive protein (≥ 0.1 mg/dl < 0.1 mg/dl) - Homeostatic model assessment-insulin resistance index (≥ 2.15, < 2.15) - Low density lipoprotein cholesterol (≥ 100 mg/dl, <100 mg/dl) - Platelets (<150 × 10 ^9^ /L, >150 × 10 ^9^ /L) - Total cholesterol (≥ 200 mg/dl, < 200 mg/dl) - Triglycerides (≥ 150 mg/dl, <150 mg/dl) - White blood cells (<4.5× 10^9^/L, ≥ 4.5× 10^9^/) |
